# Analyzing the Influence of Titanium Content in 5087 Aluminum Filler Wires on Metal Inert Gas Welding Joints of AA5083 Alloy

**DOI:** 10.3390/ma17205017

**Published:** 2024-10-14

**Authors:** Jiaolong Liu, Xin Nai, Hao Ran, Pengcheng Wang, Haiyan Chen, Xianqi Meng, Xiaojun Chen, Wenya Li, Yuzeng Chen

**Affiliations:** 1 State Key Laboratory of Solidification Processing, Northwestern Polytechnical University, No. 127 Youyi Xilu, Xi’an 710072, China; 2 Ningbo Bode Hightech Co., Ltd., Ningbo 315000, Chinaxiaojun.chen@bedra.cn (X.C.); 3 Ningbo Institute, Northwestern Polytechnical University, Ningbo 315103, China

**Keywords:** MIG welding, aluminum alloys, microstructure, mechanical properties

## Abstract

As the demand for lightweight structures in the transportation industry continues to rise, AA5083 aluminum alloy has become increasingly prominent due to its superior corrosion resistance and weldability. To facilitate the production of high-quality, intricate AA5083 components, 5087 aluminum filler wire is commonly utilized in metal inert gas (MIG) welding processes for industrial applications. The optimization of filler wire composition is critical to enhancing the mechanical properties of AA5083 MIG-welded joints. This study investigates the effects of modifying 5087 aluminum filler wires with different titanium (Ti) contents on the microstructure and weldability of AA5083 alloy plates using MIG welding. The influence of Ti contents was systematically analyzed through comprehensive characterization techniques. The findings reveal that the constitutional supercooling induced by the Ti element and the formation of Al_3_Ti facilitate the heterogeneous nucleation of α(Al), thereby promoting grain refinement. When the Ti content of 5087 filler wire is 0.1 wt.%, the grain size of the weld center was 78.48 μm. This microstructural enhancement results in the improved ductility of the AA5083 MIG-welded joints, with a maximum elongation of 16.64% achieved at 0.1 wt.% Ti addition. The hardness of the joints was the lowest in the weld center zone. This study provides critical insights into the role of Ti content in MIG welding and contributes to the advancement of high-performance filler wire formulations.

## 1. Introduction

With the rapid advancement of the transportation industry, the demand for lightweight and high-strength materials has garnered increasing attention to reduce energy and resource consumption [1,2]. AA5083 aluminum alloy, a member of the Al-Mg-Mn alloy family, is characterized by its low density, high strength, excellent corrosion resistance, and superior weldability [3,4,5]. Owing to these outstanding properties, AA5083 aluminum alloy has been widely adopted in the fabrication of welded structures for transportation vehicles, including ship hulls, tanker trucks, and pressure vessels [6,7,8].

Common industrial welding processes include fusion welding, solid-state welding, and brazing. Among these, fusion welding offers several advantages, such as ease of automation and high production efficiency [9,10,11]. The most prevalent fusion welding processes for aluminum alloy joints are metal inert gas (MIG) welding, tungsten inert gas (TIG) welding, electron beam welding (EBW), and laser beam welding (LBW) [12]. However, EBW requires a vacuum environment, necessitating expensive equipment and additional time for preparation [13]. Similarly, LBW demands costly equipment with high maintenance expenses, significantly increasing industrial production costs [14]. Additionally, the high reflectivity of aluminum alloys when encountering laser energy results in low absorption during the LBW process, leading to poor joint formation [15]. TIG welding, while effective, suffers from lower welding speeds compared to MIG welding and is unsuitable for joining thick-plate materials [16]. Consequently, considering the industrial applications of AA5083 aluminum alloy structures, MIG welding emerges as the preferred method for fabricating welded joints.

It is widely recognized that the properties of materials are significantly influenced by their microstructure [17]. To enhance the mechanical properties of MIG-welded aluminum alloy joints, various methods have been proposed to refine their microstructure through parameter optimization. Shao et al. utilized artificial neural networks based on finite element modeling to optimize welding parameters, successfully controlling the welding residual stress and deformation in MIG-welded 6061-T6 Al alloy T-joints [18]. Beyond optimizing conventional welding parameters, such as current and voltage, some researchers have explored the proper post-weld heat treatment (PWHT) is an effective method for improving welded joint performance [19]. Ye et al. successfully fabricated high-strength 7075 aluminum alloy joints using a double-pulse MIG welding process, finding that the grains in the weld zone were significantly refined due to the high-frequency oscillation of the welding current [20]. Kuang et al. investigated the effects of pulse mode and frequency on the microstructure and properties of 2219 aluminum alloy ultra-high-frequency pulse MIG-welded joints, demonstrating that the grain size in the weld zone decreased progressively with increasing ultra-high-frequency pulse frequency [21]. Additionally, the use of external magnetic fields has been shown to be an effective approach for producing robust MIG-welded aluminum alloy joints. Wu et al. developed a high-speed MIG welding technique assisted by compound external magnetic fields for 6N01-T6 aluminum alloy, which resulted in refined grains in the weld zone and enhanced both the tensile strength and impact toughness of the joints [22].

Based on the above studies, it is evident that various methods exist to enhance the mechanical properties of MIG-welded aluminum alloy joints. Despite the potential of this method, the methods mentioned above face challenges such as high costs and complex processes. Consequently, an increasing number of researchers are focusing on optimizing the composition of filler wire to improve the mechanical properties of welded joints. Pang et al. incorporated trace cerium (Ce) elements into Zn-Al brazing material [23]; the results indicated that the spreading performance and mechanical properties of the weld joint were improved, and the microstructures were refined. Currently, there are also some studies concerning the optimization of filler wire composition by incorporating elements such as zirconium (Zr), Ti, and magnesium (Mg) [24,25]; compared to other elements, Ti is more affordable and not only enhances the spreading performance of filler wire but also refines the grain size of the weld.

In this study, the MIG welding process was employed to join AA5083 aluminum alloys using 5087-aluminum filler wires modified with different Ti contents. The objective was to elucidate the role of Ti contents in the fabrication of aluminum MIG-welded joints. A systematic investigation and analysis were conducted on the interfacial microstructure and mechanical properties of the AA5083 MIG-welded joints. Through microstructure characterization and mechanical testing, the relationship between Ti contents in the filler wire, microstructure, and mechanical properties of the joints were revealed. This study offers valuable insights into the impact of Ti content in filler wires on MIG-welded joints, contributing to the development of high-quality filler wires, and enhances the use of MIG welding in sectors such as vehicle transportation.

## 2. Experimental Materials and Methods

In this paper, 5087 aluminum alloy wires were fabricated using a continuous casting direct rolling process. The raw materials included pure Al (99.99 wt.%), pure Mg (99.99 wt.%), and alloys such as Al-20Mn, Al-10Cr, Al-10Zr, and Al-6Ti. Three types of 5087 alloy filler wires with different Ti contents were prepared by calculating and adding different mass fractions of the Al-6Ti alloy. Initially, the raw materials were melted in a resistance furnace at a temperature of 780 °C, then the liquid alloy was subjected to the continuous casting direct rolling process, annealing, and drawing to produce filler wires with a diameter of 1 mm.

To ensure the complete removal of oil and oxide films from the surfaces to be welded, two AA5083 aluminum alloy plates with dimensions of 200 mm × 100 mm × 6 mm were polished with SiC sandpaper and then ultrasonically cleaned in anhydrous ethanol. The chemical composition of the aluminum alloy plates is presented in Table 1. A butt joint configuration was employed, and filler wires containing different amounts of Ti (0.03 wt.%, 0.07 wt.%, and 0.10 wt.%) were used to weld the aluminum alloy plates via MIG welding. These filler wires were designated as Filler 1, Filler 2, and Filler 3, with their respective chemical compositions also detailed in Table 1. During the welding process, the filler feeding speed, welding speed, arc current, and arc voltage were maintained at 4.20 m/min, 5.00 m/min, 92 A, and 12.1 V, respectively.

Following the welding process, the joints were sectioned into specimens measuring 30 mm × 5 mm × 6 mm for microstructural characterization, as well as dog-bone samples for mechanical property evaluation. A scanning electron microscope (SEM, Verios G4), equipped with energy-dispersive spectroscopy (EDS) and electron backscatter diffraction (EBSD) detectors, was employed to analyze the microstructure, chemical composition, and fracture morphology of the AA5083 MIG-welded joint. The mechanical properties of the joints were assessed through tensile testing, utilizing a universal testing machine. The tensile strength reported represents the average of three measurements. Furthermore, microhardness testing was conducted across the weld seam cross-section at 0.2 mm intervals, applying a load of 300 g for 15 s.

## 3. Results and Discussion

### 3.1. The Interfacial Microstructure of Joints

**Figure 1:**
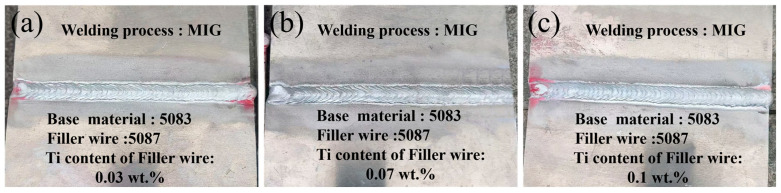
Macrographs of AA5083 MIG-welded joints prepared by different filler wires: (**a**) Filler 1, (**b**) Filler 2, and (**c**) Filler 3.

Figure 1 presents the macroscopic morphology of the weld on the 5083 aluminum alloy MIG-welded joints produced using Fillers 1, 2, and 3 under the parameters outlined in the previous section. It is evident that, under these MIG welding conditions, the macroscopic appearance of the joint is of high quality, with no significant welding defects such as spatter, undercut, incomplete fusion, or cracks observed on the weld. Additionally, the macrostructure analysis reveals a distinct fish-scale pattern on the weld surface, with a uniform width and well-defined ripples, suggesting that when welding 5083 aluminum alloys using the MIG process, both the current (92 A) and voltage (12.1 V) remain very stable and largely unchanged throughout the process, while the filler wire feeding speed was steady and balanced, contributing to the uniform formation of the fish-scale pattern.

**Figure 2:**
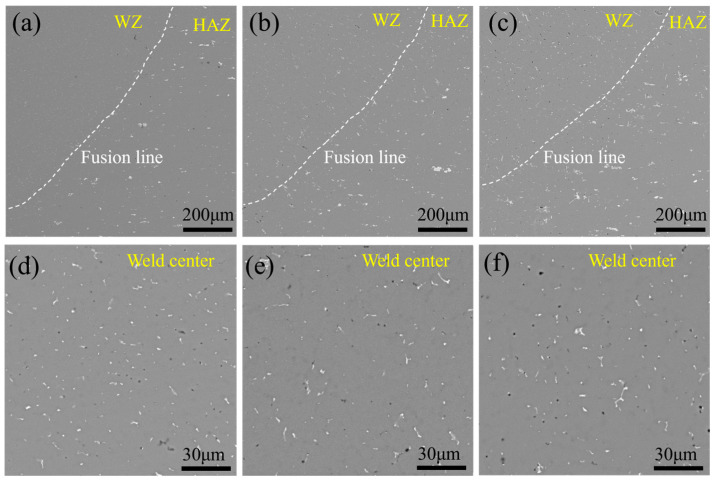
Microstructures of different regions in AA5083 MIG-welded joints fabricated by different filler wires: (**a**,**d**) Filler 1, (**b**,**e**) Filler 2, and (**c**,**f**) Filler 3.

Following the characterization of the AA5083 MIG-welded joints produced with Ti-modified 5087 aluminum filler wires, this study examines the influence of the Ti content in the filler wires on the microstructure of the joints. Figure 2 illustrates the microstructure of different regions of the welded joint; the dashed white line drawn in Figure 2 represents the fusion line of the AA5083 MIG-welded joints. As depicted in Figure 2, numerous black and white precipitate phases are formed and uniformly distributed in the matrices of the AA5083 MIG-welded joints fabricated with different filler wires. Notably, on the side closer to the weld zone (WZ), the dispersions of the dark and white second-phase particles are smaller compared to the second-phase particles present on the side adjacent to the heat-affected zone (HAZ). The difference in size between the black and white precipitate phases from different regions is attributed to the influence of thermal cycling during the welding process, which leads to the formation of a larger second phase in the HAZ [26,27]. Figure 2d–f display the distributions of the second phase in the weld centers of welded joints made with different 5087 filler wires. From Figure 2d–f, it is evident that there is no significant change in the size of the second phase in the weld center as the Ti content increases.

**Figure 3:**
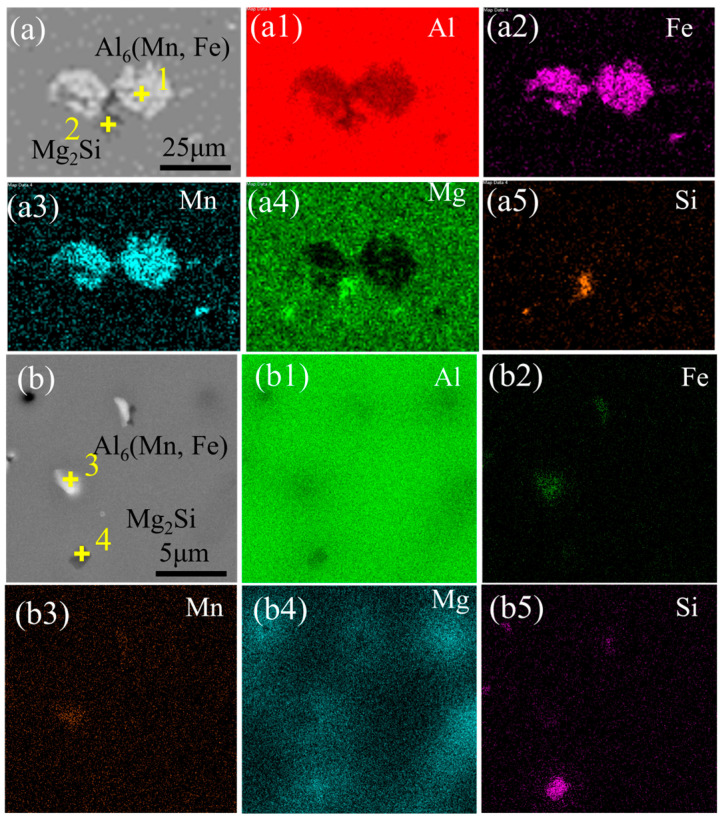
EDS analysis of different regions in the AA5083 MIG-welded joint produced with Filler 3: (**a**) HAZ, (**a1**) Al, (**a2**) Fe, (**a3**) Mn, (**a4**) Mg, (**a5**) Si, (**b**) WZ, (**b1**) Al, (**b2**) Fe, (**b3**) Mn, (**b4**) Mg, (**b5**) Si.

Figure 3 illustrates the EDS analysis of the black and white precipitate phases in WZ and HAZ in the AA5083 MIG-welded joint produced with Filler 3. The elemental composition at the yellow crosses (1–4) in Figure 3 is presented in Table 2. As shown in Figure 3, the white phase in both the HAZ and WZ consists primarily of Al, Fe, and Mn elements, and the atomic ratio of Al, Fe, and Mn is approximately 6:1:1. Using previous research findings [28], it can be determined that these precipitate phases are predominantly Al_6_(Fe, Mn). The black phase in both the HAZ and the WZ consists primarily of Mg and Si elements, with an atomic ratio close to 2:1; according to the studies conducted by Zhang [29] and Yang [30], these dark phases are presumed to be Mg_2_Si phases. One of the aluminum elements in the dark phases should be identified within the matrix.

**Figure 4:**
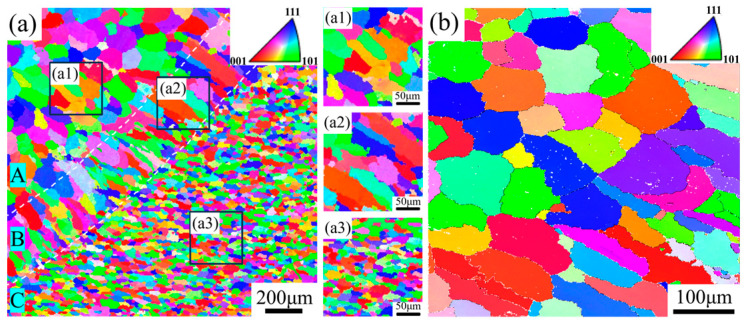
Grain morphology in the different zones of the AA5083 MIG-welded joint fabricated by Filler 3: (**a**) the zones near the fusion line, A (**a1**) the dendritic grain region, B (**a2**) the columnar grain region, C (**a3**) the equiaxed grain region, and (**b**) the heat-affected zone.

Figure 4 presents the EBSD analysis of different zones in the WZ of the weld joint produced with Filler 3. The fusion line, representing the weld contour visible in the cross-section, delineates the boundary between the weld zone and the base material. It is generally accepted that the formation of different microstructures is linked to the solidification mode, which is determined by the constitutional supercooling [31]. As illustrated in Figure 4a, distinct transition regions are evident near the fusion line, which can be categorized into three zones, Zone A, Zone B, and Zone C, based on grain size. Table 3 demonstrates the average grain sizes of different regions from the weld joint produced with Filler 3. Zone A is characterized by dendritic structures with an average grain size of 73.3 μm, formed as a result of significant compositional undercooling at the weld center, which promotes dendritic growth. Zone B consists of columnar grains with an average grain size of 65.8 μm, attributed to the rapid heat dissipation perpendicular to the fusion line, where the temperature gradient is highest, fostering the development of columnar grains along the weld periphery. Zone C is predominantly composed of fine equiaxed grains with an average grain size of 32.4 μm, generated by the substantial undercooling that occurs when molten weld droplets contact the base material, leading to the formation of numerous, uniformly distributed fine equiaxed grains. From Figure 4a, it is evident that the grain size gradually increases from region C to region A. This increase is attributed to the gradual decrease in supercooling experienced by the alloy as it moves from region C to region A. The EBSD analysis in Figure 4b reveals the grain morphology in the HAZ, where coarser grains with an average size of 68.3 μm are observed. This is primarily due to the high thermal conductivity of aluminum alloys, which facilitates the rapid dissipation of heat to adjacent areas during welding. Consequently, the elevated temperature in the HAZ promotes grain growth, resulting in a coarser microstructure [32].

### 3.2. The Effects of Ti Content on the Microstructure of Joints

**Figure 5:**
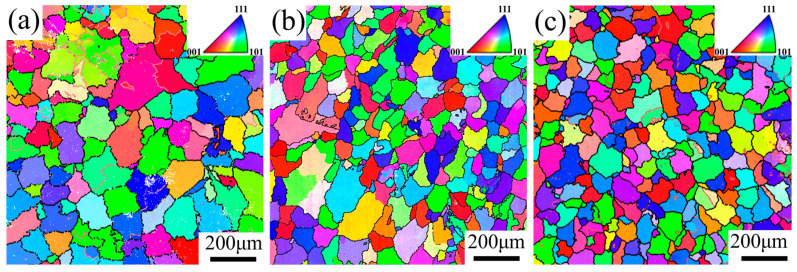
Grain morphology in the welding center zone of the AA5083 MIG-welded joints prepared by different filler wires: (**a**) Filer 1, (**b**) Filler 2, and (**c**) Filler 3.

Figure 5 illustrates the grain morphology within the WZ center of AA5083 MIG-welded joints fabricated with different filler wires, and Table 4 demonstrates the average grain size at the WZ center of AA5083 MIG-welded joints prepared by different filler wires. The grain sizes for the MIG-welded joints prepared with Filler 1, Filler 2, and Filler 3 are 113.74 μm, 86.99 μm, and 78.48 μm, respectively. As the Ti content in the filler wires increases, the grain size in the weld center zone decreases. The EDS results of the WZ center in Figure 3 indicate that the Ti element primarily exists as a solid solution within the matrix and is uniformly distributed throughout it. Based on previous studies [33], we can attribute the Ti refinement of weld grain to two aspects. On the one hand, according to solute theory [34], Ti leads to compositional supercooling during solidification and promotes the heterogeneous nucleation of α(Al). And on the other hand, the addition of trace amounts of Ti can lead to the formation of diffuse Al_3_X phases within the matrix that are not detectable by EDS [35]. They closely resemble the crystal structure of the α(Al) matrix, and serve as effective heterogeneous nucleation cores for α(Al) [36], which explains the refinement of grains in the WZ center as the content of Ti increases.

### 3.3. The Mechanical Properties of AA5083 MIG-Welded Joints

**Figure 6:**
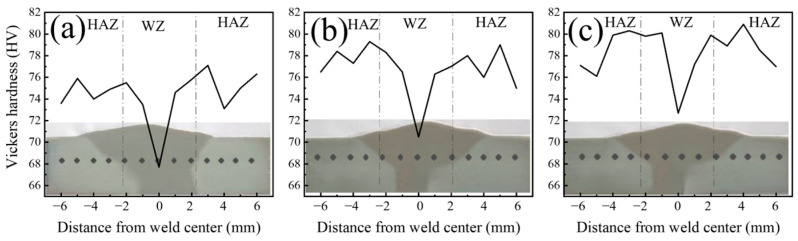
Hardness distribution of AA5083 MIG-welded joint prepared by different filler wires: (**a**) Filer 1, (**b**) Filler 2, and (**c**) Filler 3.

The microstructure of MIG-welded joints evolves with increasing Ti content in the filler wires, which subsequently influences their mechanical properties. Figure 6 illustrates the hardness distribution of AA5083 MIG-welded joints fabricated with different filler wires. The dotted line in the Figure 6 shows the dividing line between the HAZ and the WZ. The hardness distribution is symmetrical across the weld zone and the HAZ, with the lowest hardness value observed in the weld zone center. Welding involves the solidification of the alloy, with the center of the weld being the last area to solidify. This region often exhibits various defects, such as segregation and porosity, making it the weakest in the welded joint, resulting in the lowest hardness. Furthermore, a comparison of the hardness test results reveals that the hardness values of the weld zones in the joints prepared with Filler 2 and Filler 3 are similar, approximately 70.5 and 72.7 HV. However, the hardness in the weld zone of the joint fabricated with Filler 1 is noticeably lower, at only 67.7 HV. This can be attributed to the finer grain structure associated with the addition of Ti to the weld zones of the joints fabricated by Filler 2 and Filler 3 [37].

**Figure 7:**
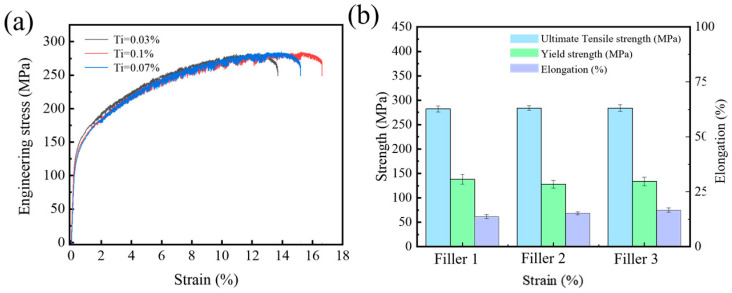
The stress–strain curve and tensile properties of the AA5083 MIG-welded joints prepared by 5087 aluminum welding wires with different Ti contents: (**a**) stress–strain curve, (**b**) tensile properties.

Figure 7 presents the stress–strain curves of the AA5083 MIG-welded joints prepared using 5087 aluminum filler wires with different Ti contents. After tensile testing, fractures in all MIG-welded joints occurred in the HAZ. The stress–strain curves exhibit similar profiles, and there are minimal differences in ultimate tensile strength (UTS), yield strength (YS), and elongation, as shown in Table 5. Meanwhile, the numerical variations in the YS and UTS of the welded joints prepared by different filler wires fall within the error range observed in several sets of experiments conducted during this study. Therefore, based on the experimental results, it can be concluded that adding 0.03 wt.%, 0.07 wt.%, and 0.1 wt.% Ti to 5087 aluminum alloy filler wires does not have much impact on the UTS and YS of the welded joints. However, it is noteworthy that as the Ti content in the filler wire increases, there is a slight increase in elongation. When the filler wire with 0.1 wt.% Ti content (Filler 3) is utilized, the maximum elongation of 16.64% is reached. The slight increase in elongation is attributed to the refinement of grains in the weld zone. The smaller and more uniformly distributed grains in the weld zone contribute to improving the ductility [38].

**Figure 8:**
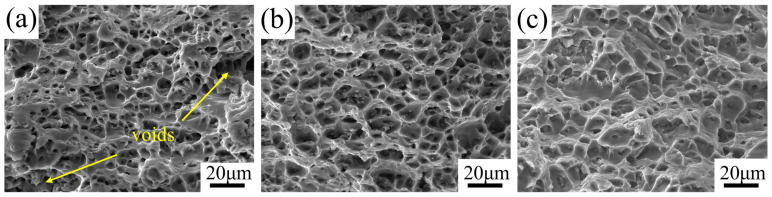
Tensile fracture morphologies of AA5083 MIG-welded joints prepared by different filler wires: (**a**) Filer 1, (**b**) Filler 2, and (**c**) Filler 3.

Figure 8 displays the tensile fracture surfaces of the MIG-welded joints fabricated using 5087 aluminum filler wires with different Ti contents. Numerous dimples are evident on the fracture surfaces of all joints, indicating a ductile fracture mode. Notably, the fracture surface of the joint produced with Filler 1 exhibits larger pores, suggesting an increased stress concentration, which reduces the effective load-bearing area and consequently lowers ductility. In contrast, the fracture surfaces of the joints fabricated with Fillers 2 and 3 display much smaller dimples. As discussed earlier, finer grain structures inhibit dislocation movement, leading to reduced stress concentration [39]. Consequently, when a fracture occurs, the dimples are smaller and more uniformly distributed.

## 4. Conclusions

The successful bonding of AA5083 aluminum alloys was achieved using A5087 aluminum filler wires with different Ti contents. Through detailed characterization, the microstructure and mechanical properties of AA5083 MIG-welded joints were systematically investigated, elucidating the effects of Ti content in the filler wires on the resulting welds. Based on our characterization and in-depth analysis, the following key conclusions can be drawn:

(1) The AA5083 MIG-welded joints fabricated using the three Ti-containing filler wires exhibited sound metallurgical bonding and were free from defects. In both the weld zone and the heat-affected zone, Al_6_(Fe, Mn), and Mg_2_Si precipitate phases were observed.
(2) When AA5083 aluminum alloy plates were MIG-welded with filler wires containing higher Ti content, the constitutional supercooling induced by the Ti element and the formation of Al_3_Ti facilitates the heterogeneous nucleation of α(Al), thereby promoting grain refinement.
(3) The refinement of grains in the weld zone, resulting in a higher density of grain boundaries, hindered dislocation movement during tensile testing, thereby enhancing the ductility of the joints. When MIG welding was conducted using filler wire containing 0.1 wt.% Ti, the joint exhibited the highest elongation, reaching 16.64%.

## Figures and Tables

**Table 1:** Chemical compositions of filler wires and base material in this study.

Materials	Mg	Mn	Fe	Cr	Si	Zr	Ti	Al
Base materials	4.7	0.87	0.12	0.07	0.04	-	0.10	Bal.
Filler 1	4.9	0.87	0.11	0.08	0.04	0.1	0.03	Bal.
Filler 2	4.8	0.86	0.11	0.08	0.04	0.1	0.07	Bal.
Filler 3	4.8	0.87	0.12	0.08	0.04	0.1	0.10	Bal.

**Table 2:** EDS point results (at.%) in Figure 3.

Position	Mg	Mn	Ti	Si	Fe	Al
Point 1	0.52	14.38	0.01	1.22	20.90	Bal
Point 2	10.80	0.38	0.00	4.93	0.02	Bal
Point 3	0.14	10.23	0.05	0.12	8.39	Bal
Point 4	9.8	0.54	0.04	4.75	0.39	Bal

**Table 3:** The average grain sizes of different regions from the weld joint produced with Filler 3.

Region	A	B	C	Heat-Affected Zone
Grain size/(μm)	73.3	65.8	32.4	68.3

**Table 4:** Grain size at the center of the AA5083 MIG-welded joints prepared by different filler wires.

Weld Joint	Filler 1	Filler 2	Filler 3
Grain size/(μm)	113.74	86.99	78.48

**Table 5:** Tensile test results for AA5083 MIG-welded joints prepared by different filler wires.

Filler Wire	Ultimate Tensile Strength (MPa)	Elongation (%)	Yield Strength (MPa)
1	282.2	13.69	136.88
2	284	15.21	128.93
3	284	16.64	133.57

## Data Availability

The original contributions presented in the study are included in the article. further inquiries can be directed to the corresponding authors.
